# Supplementary material for: A Systematic Review and Meta-Analysis of the Pathology Underlying Aneurysm Enhancement on Vessel Wall Imaging
Source: Int J Mol Sci. 2024 Feb 26;25(5):2700. doi: 10.3390/ijms25052700 (PMC10931983; doi:10.3390/ijms25052700)
Supplement: Supplementary file 1 [file ijms-25-02700-s001.zip › ijms-2746823-supplementary.pdf]

Supplement

Supplementary table 1

PubMed search performed in July 2021 with no date restrictions

1	MRI vessel wall imaging OR MRI VWI OR VWI
2	intracranial aneurysm or cerebral aneurysm or aneurysm
3	pathology or histopathology
4	human or homo sapiens
5	NOT (Aortic or abdominal or animal)
6	1 and 2 and 3 and 4 and 5

Embase search performed in July 2021 with no date restrictions

1	MRI vessel wall imaging OR MRI VWI OR VWI
2	intracranial aneurysm or cerebral aneurysm or aneurysm
3	pathology or histopathology
4	human or homo sapiens
5	NOT (Aortic or abdominal or animal)
6	1 and 2 and 3 and 4 and 5

Supplemental figures

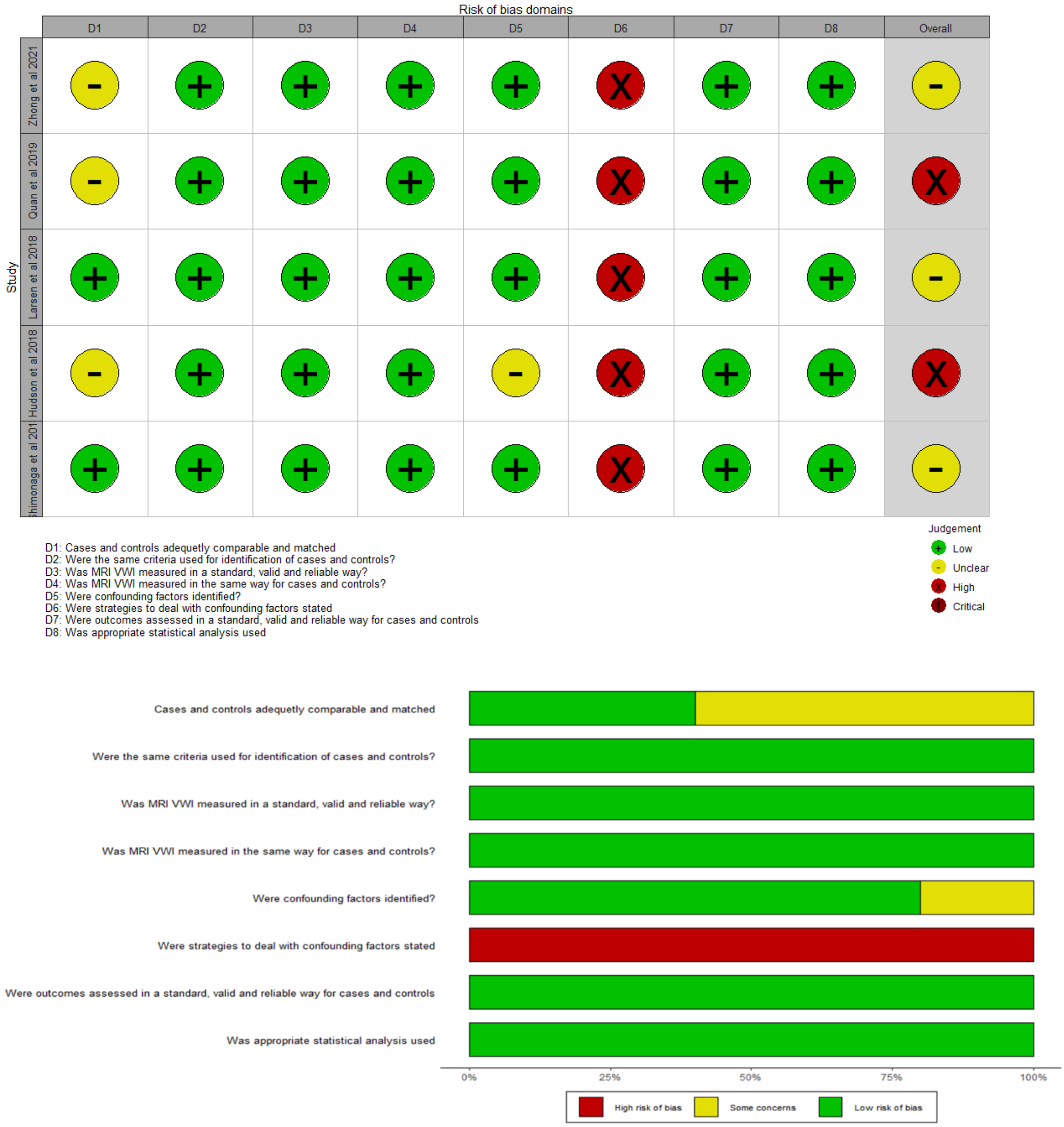

Supplemental Figure 1: Risk of bias assessments using JBI case-control checklist. The studies included are Zhong et al 2021, Quan et al 2019, Larsen et al 2018, Hudson et al 2018, Shimonaga et al 2018. The responses of yes, unclear, and no have been changed to low risk of bias, some concerns/unclear and high risk of bias respectively

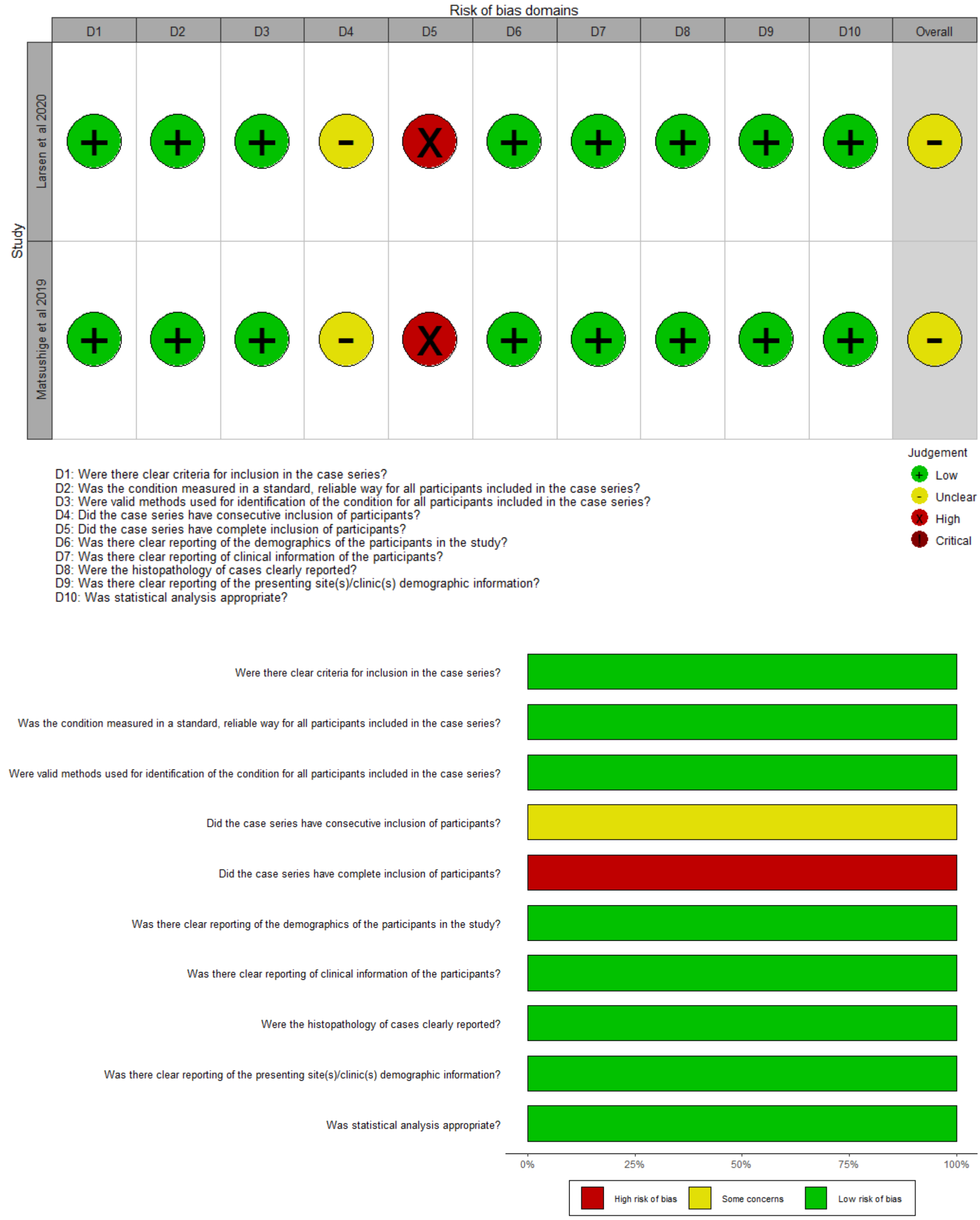

Supplemental Figure 2: Risk of bias assessments using JBI case series checklist. The studies included are Larsen et al 2020, Matsushige et al 2019. The responses of yes, unclear, and no have been changed to low risk of bias, some concerns/unclear and high risk of bias respectively
